# Admission temperature of very low birth weight infants and outcomes at three years old

**DOI:** 10.1038/s41598-022-15979-w

**Published:** 2022-07-13

**Authors:** Shin Kato, Osuke Iwata, Sachiko Iwata, Takaharu Yamada, Kennosuke Tsuda, Taihei Tanaka, Shinji Saitoh

**Affiliations:** 1 Department of Pediatrics and Neonatology, Nagoya City University Graduate School of Medical Sciences, 1 Kawasumi, Mizuho-cho, Mizuho-ku, Nagoya, 467-8601 Japan; 2 Department of Pediatrics, Japanese Red Cross Aichi Medical Center Nagoya Daini Hospital, Nagoya, Japan

**Keywords:** Intrauterine growth, Neonatal brain damage

## Abstract

The lower body temperature of preterm newborns at admission to neonatal intensive care units (NICUs) is inversely associated with their morbidities and mortalities before discharge. This retrospective cohort study aimed to determine whether admission rectal temperature in very low birth weight infants (VLBWIs) is independently associated with a composite outcome of death or moderate-to-severe neurodevelopmental impairments as defined by a performance developmental quotient of < 70 at three years of age. VLBWIs admitted to the NICU between April 2010 and March 2016 were assesed. Developmental assessment was completed in 216 newborns. Nine and two infants died before and after discharge, respectively. A higher admission temperature was associated with a lower incidence of death or moderate-to-severe neurodevelopmental impairments with adjustment for gestational age, sex, antenatal steroid use, Apgar score, severe intraventricular hemorrhage, and severe bronchopulmonary dysplasia (odds ratio [OR] 0.424; 95% confidence interval [CI] 0.250–0.717; p = 0.001). The admission temperature remained as an independent variable of adverse outcome at three years of age even when the study cohort was limited to surviving infants (OR 0.448; 95% CI 0.259–0.774; p = 0.004). Further studies are needed to assess whether avoiding low body temperature at admission results in better long-term neurodevelopmental outcomes in VLBWIs.

## Introduction

The lower body temperature of preterm newborns at admission to neonatal intensive care units (NICUs) is inversely associated with their morbidities and mortalities before discharge^1–3^. Therefore, maintaining a body temperature of 36.5 °C to 37.5 °C is recommended when resuscitating preterm infants^4^. Despite an increasing concern for body temperature maintenance during resuscitation after birth, more than one-third of preterm infants still experience a low admission temperature of < 36.5 °C even in developed countries^3,5,6^. Further, insufficient clinical evidence exists regarding whether low admission temperature at birth is associated with short-term outcomes only or has extensive influence on long-term neurodevelopmental outcomes. Ting et al. assessed the relationship between the admission temperatures (rectal, skin, or axillary) of preterm infants (born at less than 29 weeks’ gestation) and their outcomes using a dataset from a large-scale registry. They found that lower admission temperatures (< 36.5 °C) were associated with the composite outcome of death or significant neurodevelopmental impairment at 18 to 21 months of corrected age (odds ratio [OR] 1.05; 95% confidence interval [CI] 1.05–1.66)^7^. However, of a number of secondary outcomes, only severe language delay showed a weak relationship with admission hypothermia (OR 1.58; 95% CI 1.03–2.42), leaving uncertainty around whetehr admission hypothermia is associated with both short-term mortality and long-term neurodevelopmental impairments. The lack of robust relationships between admission temperature and outcomes in this register-based study might be attributed to the heterogeneity of the patient background, location of birth, temperature management, and location of temperature monitoring. Recent studies have suggested that short-term outcomes of preterm infants have improved to a convincingly high level in developed countries^8,9^. However, if admission temperature in preterm infants is associated with their long-term neurodevelopmental outcomes, the strategy during neonatal resuscitation of maintaining the body temperature may require further improvement.

This study aimed to determine whether admission rectal temperature in very low birth weight infants (VLBWIs) is associated with a long-term composite outcome of death or moderate-to-severe neurodevelopmental impairment at three years of age.

## Results

**Figure 1:**
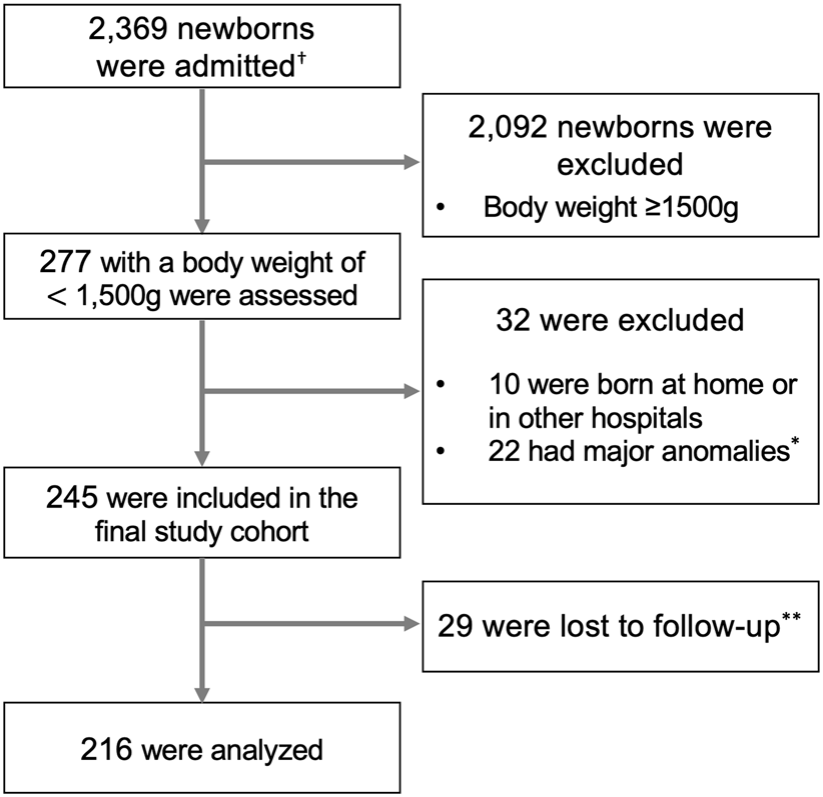
Flow diagram of the study population. ^†^Newborns admitted to the NICU between April 2010 and March 2016. *Newborns excluded due to major congenital anomalies or chromosomal aberrations. **Twenty-nine newborns were lost to follow-up due to relocation (n = 22) and unknown reasons (n = 7).

**Figure 2:**
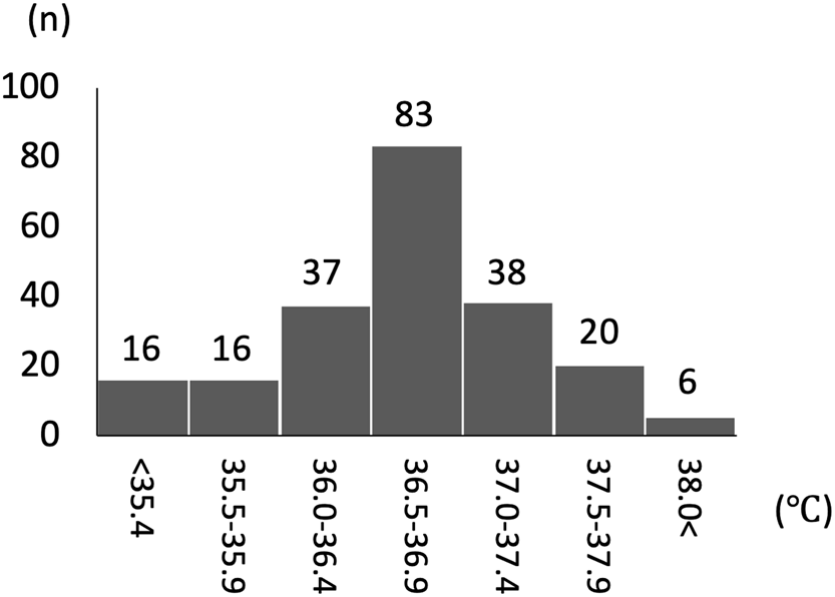
Histogram depicting the distribution of admission temperature of the study population. Rectal temperature was measured within 5 min of admission. Among the participants, 31.9% (n = 69) of the newborns had an admission temperature of < 36.5 °C.

Of the 245 newborns in the final study cohort, 29 (11.8%) were lost to follow up. The remaining 216 newborns were born after 28.8 (3.4) weeks of gestation and 1040 (315) g at birth (Fig. 1 and Table 1). The cohort included 37 (17%) infants born at < 25 weeks of gestation, and 87 (40%) infants born between 25 and 28 weeks of gestation. The mean admission temperature of the whole cohort was 36.6 (0.8)°C; 31.9% (n = 69) of the included newborns had an admission temperature of < 36.5 °C (Fig. 2). Nine and two infants died before and after discharge, respectively. Among the survivors up to three years of age (n = 205), 60 children had a low performance developmental quotient (DQ) of < 70 at three years of age.

**Table 1:** Characteristics of the study population.

	All newborns (n = 216)		Subgroups with admission temperature			
			< 36.5 °C (n = 69)		36.5 °C ≤ (n = 147)	
**Antenatal variables**						
Chorioamnionitis	46	(21.3%)	18	(26.1%)	28	(19.0%)
Antenatal steroids	134	(62.0%)	36	(52.2%)	98	(66.7%)
Multiple births	60	(27.8%)	10	(14.5%)	50	(34.0%)
Cesarean section	197	(91.2%)	59	(85.5%)	138	(93.9%)
**Variables at birth**						
Gestational age (week)	29	(3.4)	29	(4.3)	29	(2.9)
Birth weight (g)	1040	(315)	941	(334)	1086	(296)
Female sex	103	(47.7%)	37	(53.6%)	66	(44.9%)
5-min Apgar score < 5	28	(13%)	13	(19.1%)	15	(10.2%)
Surfactant administration	187	(86.6%)	57	(82.6%)	130	(88.4%)
Resuscitation time (min)	24.7	(11.6)	24.0	(9.0)	25.0	(12.6)
Admission temperature (°C)	36.6	(0.8)	35.8	(0.7)	37.0	(0.4)
**Short-term outcomes**						
Ductus arteriosus*	110	(50.9%)	32	(47.1%)	78	(53.1%)
Culture positive sepsis	26	(12.0%)	10	(14.7%)	16	(10.1%)
Intraventricular hemorrhage**	20	(9.3%)	6	(8.7%)	14	(9.5%)
Intestinal perforation^†^	13	(6.0%)	6	(8.7%)	7	(4.8%)
Bronchopulmonary dysplasia^††^	98	(45.4%)	34	(51.5%)	64	(44.8%)
Death before discharge	9	(4.2%)	5	(7.2%)	4	(2.7%)
Death within 7d of life	2	(0.9%)	2	(2.9%)	0	(0%)
**Outcomes at age three years**						
Death after discharge	2	(0.9%)	1	(1.4%)	1	(0.7%)
Death or developmental quotient < 70	71	(32.9%)	30	(43.5%)	41	(27.9%)

Data are presented as number (percentages) or mean (standard deviations).

*Hemodynamically symptomatic ductus arteriosus requiring pharmacological or surgical intervention.

**Grade III/VI by Papile’s definition.

^†^Any type of perforation included.

^††^Severe bronchopulmonary dysplasia defined by The National Institute of Child Health and Human Development 2001.

The univariate analysis showed that smaller gestational age (p < 0.001), singleton (p = 0.006), low Apgar scores (p = 0.004), symptomatic patent ductus arteriosus (p = 0.003), septicemia (p = 0.001), severe intraventricular hemorrhage (IVH) (p < 0.001), severe bronchopulmonary dysplasia (BPD) (p = 0.001), and lower admission temperature (p < 0.001) were associated with worse primary outcomes (Table 2). In the multivariate model, a higher admission temperature was associated with a lower incidence of adverse primary outcomes, with adjustment for gestational age, sex, antenatal steroid use, Apgar score, severe IVH, and severe BPD (OR 0.424; 95% CI 0.250, 0.717; p = 0.001; Table 3; See online supplementary Table S1 for an alternative model). The secondary analysis in survivors consistently identified lower admission temperature as an independent variable of moderate-to-severe neurodevelopmental impairment at three years of age (Table 4; OR 0.448; 95% CI 0.259–0.774; p = 0.004).

**Table 2:** Independent variables of death or neurodevelopmental impairment (developmental quotient [DQ] < 70) at three years of age.

	Odds ratio			p
	Mean	95% CI		
		Lower	Upper	
Gestational age (week)	0.753	0.678	0.836	< 0.001
Female sex	0.608	0.341	1.082	0.091
Antenatal corticosteroid	0.764	0.428	1.365	0.364
Multiple births	0.359	0.173	0.745	0.006
Cesarean delivery	0.404	0.156	1.044	0.061
5-min Apgar score < 5	3.259	1.446	7.346	0.004
Resuscitation time (min)	0.972	0.931	1.014	0.191
Admission temperature (°C)	0.458	0.300	0.698	< 0.001
Ductus arteriosus*	2.426	1.341	4.387	0.003
Culture positive sepsis	4.000	1.708	9.366	0.001
Intraventricular hemorrhage**	14.901	4.198	52.889	< 0.001
Intestinal perforation^†^	2.534	0.819	7.843	0.107
Bronchopulmonary dysplasia^††^	2.752	1.496	5.064	0.001

*Hemodynamically symptomatic ductus arteriosus requiring pharmacological or surgical intervention.

**Grade III/VI by Papile’s definition.

^†^Any type of perforation included.

^††^Severe bronchopulmonary dysplasia defined by The National Institute of Child Health and Human Development 2001.

**Table 3:** Multivariate model explaining death or neurodevelopmental impairment at three years of age.

	Odds ratio			p
	Mean	95% CI		
		Lower	Upper	
Gestational age in weeks	0.771	0.661	0.899	0.001
Female sex	0.490	0.245	0.982	0.044
Antenatal corticosteroid	0.723	0.354	1.474	0.372
5-min Apgar score < 5	0.774	0.257	2.334	0.649
Admission temperature (°C)	0.424	0.250	0.717	0.001
Intraventricular hemorrhage*	11.680	2.769	49.273	< 0.001
Bronchopulmonary dysplasia**	0.720	0.284	1.823	0.487

Neurodevelopmental impairment is defined as a performance developmental quotient of < 70.

*Grade III/VI by Papile’s definition.

**Severe bronchopulmonary dysplasia defined by The National Institute of Child Health and Human Development 2001.

**Table 4:** Multivariate model explaining neurodevelopmental impairment in survivors to the three years of age.

	Odds ratio			p
	Mean	95% CI		
		Lower	Upper	
Gestational age in weeks	0.811	0.690	0.954	0.011
Female sex	0.524	0.258	1.063	0.073
Antenatal corticosteroid	0.732	0.354	1.512	0.399
5-min Apgar score < 5	0.944	0.306	2.908	0.920
Admission temperature (°C)	0.448	0.259	0.774	0.004
Intraventricular hemorrhage*	10.444	2.388	45.678	0.002
Bronchopulmonary dysplasia**	0.812	0.318	2.069	0.662

Neurodevelopmental impairment is defined as a performance developmental quotient of < 70.

*Grade III/VI by Papile’s definition.

** Severe bronchopulmonary dysplasia defined by The National Institute of Child Health and Human Development 2001.

## Discussion

Building on previous studies, our study confirmed that admission rectal temperature in VLBWIs is associated with long-term composite outcomes of death or moderate-to-severe neurodevelopmental impairments at three years of age. Furthermore, an association was consistently observed between admission temperature and neurodevelopmental impairments in the secondary analysis of the survivors.

Since Silverman et al. first reported the benefit of using relatively higher ambient temperature to reduce mortality in preterm infants^10^, other studies have identified associations between admission temperature and short-term adverse events, such as in-hospital death and neonatal complications^1,2,5,7^. Hence, clinical guidelines for neonatal resuscitation have emphasized the importance of body temperature monitoring in preterm infants from the early period of establishing the resuscitation programs^11^. Although the incidence of low admission temperature and related short-term outcomes of preterm infants has improved^1,12,13^, the question remains of whether the admission temperature is associated with long-term outcomes of preterm infants despite excluding the influence of short-term morbidities. In a study of more than 2700 registered preterm neonates < 29 weeks gestation, Ting et al. reported that the admission temperature measured at various locations showed a modest relationship with the composite outcome of death or significant neurodevelopmental impairments^7^. However, the relationship was relatively less robust compared to the large sample size, and the composite outcome was mostly contributed by mortality. In our study, we aimed to investigate whether admission core temperature is associated with the outcome in a high-survival cohort of VLBWIs, who were born at a single tertiary NICU and provided with similar temperature management after birth. Subsequently, admission temperature showed robust associations with the outcome of VLBWIs, even when the cohort was restricted to those who survived to the three years of age, suggesting the importance of early temperature management of VLBWIs to improve their survival and long-term neurodevelopmental outcomes.

Our current findings support the potential benefit of relatively more rigorous maintenance of body temperature during the resuscitation of preterm infants. However, the mechanisms behind adverse long-term outcomes in preterm infants due to hypothermia shortly after birth remain unclear. In preterm infants, hypothermia is linked to increased oxygen consumption and energy depletion, metabolic acidosis, surfactant deficiency, and pulmonary hypertension^14,15^. In addition, failure in the transition to the extrauterine environment, and subsequent requirement for prolonged intensive care might lead to worse neurodevelopmental outcomes in the affected newborns^16,17^. In term-born infants, complex relationships between the body temperature and neurodevelopmental outcomes have been reported. In asphyxiated infants, therapeutic hypothermia commenced within 6 h of birth could ameliorate death and improve neurodevelopmental outcomes by 18 months of corrected age^18^. However, recent studies have highlighted a paradoxical relationship between low admission temperature and adverse short- and long-term outcomes of asphyxiated newborns^19,20^. The authors of these studies speculated that reduced thermogenesis, following the most severe hypoxia–ischemia, resulted in low admission temperature and adverse outcomes in affected infants. Considering this, in our current study, newborns who experienced relatively greater difficulties in birth transition might have required more extended resuscitation, possibly leading to greater heat loss and adverse short- and long-term outcomes. In contrast to the results of previous studies, our study found that the duration of resuscitation time was not associated with low admission temperature and the primary outcome in our current dataset. Prospective studies are required to determine whether improved insulation of preterm infants after birth ameliorates their long-term outcomes.

### Strengths and limitations

Our study population was a high survival cohort, which is typical for Japanese tertiary NICUs^21^. Although the cohort size was relatively small, the follow-up rate to three years of age was 88.2%, allowing the assessment of the primary and secondary analyses in > 200 newborns. The cohort included 57% of extremely low gestational age newborns born at < 28 weeks of gestation. Considering that these newborns are most susceptible to admission hypothermia, morbidity, and mortality, and that the resuscitation strategy is not fully established for these vulnerable newborns, our study cohort might be most suitable for investigating the relationship between admission temperature and long-term outcomes. Additionally, body temperature at admission was measured rectally, which is more reliable than peripheral measurement^22^. Finally, this study was based on a retrospective analysis of prospectively collected data from a single center. The study period of over six years is relatively long, during which the treatment strategy and subsequent outcomes of newborns might change.

## Conclusion

Higher admission temperature was associated with better outcomes at three years of age in VLBWIs, which was consistently observed even when the study cohort was limited only to surviving infants. Further studies are needed to assess whether avoiding low body temperature at admission leads to better long-term neurodevelopmental outcomes, as well as reduced short-term complications.

## Methods

### Study design

This was a retrospective analysis of clinical data that were prospectively accumulated from newborns hospitalized at a tertiary NICU of the Japanese Red Cross Nagoya Daini Hospital, Nagoya, Japan, as a part of a domestic follow-up program for high-risk newborns.

### Study population

Among 2369 newborns admitted to the NICU between April 2010 and March 2016, 277 were VLBWIs (< 1500 g) (Fig. 1). We excluded newborns who were transported to the NICU after birth (n = 10) and those with congenital heart disease (n = 9), major congenital anomalies (n = 5), or chromosomal aberrations (n = 8), leaving 245 newborns in the final study cohort.

### Data collection

Maternal and patient characteristics, short-term (in-hospital), and long-term outcomes were collected from the medical records. Resuscitation time was calculated as the duration between birth and admission to the NICU. Symptomatic ductus arteriosus was defined as hemodynamically symptomatic ductus arteriosus requiring therapeutic doses of indomethacin infusion (excluding prophylactic use) or surgical closure. Grade III/IV IVH was defined using the criteria defined by Papile et al.^23^ Necrotizing enterocolitis was defined by modified Bell’s stage IIA or greater^24^. Severe BPD was defined as a requirement of > 30% oxygen or positive pressure at 36 weeks postmenstrual age^25^.

### Routine resuscitation procedure

In this hospital, approximately 90% of VLBWIs are delivered via Cesarean section. All resuscitation procedures were performed in accordance with the Neonatal Cardiopulmonary Resuscitation program of Japan^26^, which is based on the Consensus on Science with Treatment Recommendations, published by the International Liaison Committee on Resuscitation^27^. After birth, the infants were kept warm using a plastic wrap and radiant heater. Rectal temperature was routinely recorded on admission to the NICU.

### Outcomes

The primary outcome was a composite outcome of death or moderate-to-severe neurodevelopmental impairment, defined by a performance DQ of < 70 at three years of age. The DQs were assessed by experienced child psychologists using the Kyoto Scale of Psychological Development 2001, which correlates linearly with those from the Bayley Scales of Infant and Toddler Development, Third Edition^28^. The secondary outcome was moderate-to-severe neurodevelopmental impairment in survivors to the age of three years (Supplementary Table S1).

### Data analysis

Values are shown as the mean (standard deviation) unless otherwise specified. Multiple missing value imputations were performed to reduce the attrition biases due to missing data (n = 5 imputations) based on the correlation between variables with missing values and other subject characteristics. The crude effects of antenatal and postnatal variables on outcomes were assessed using univariate logistic regression analysis. The dependence of primary and secondary outcomes on admission temperature was assessed using multivariate logistic regression analysis. Known independent variables of the outcome for preterm infants were used as a priori covariates, including gestational age, sex, antenatal steroid use, 5-min Apgar score, severe IVH, and severe BPD. Statistical analysis was performed using the IBM SPSS Statistics package version 25.0 (IBM, Armonk, New York, USA).

### Ethics approval

The study protocol was approved by the Institutional Review Board of the Japanese Red Cross Nagoya Daini Hospital (#1344S). Parental informed consent was waived by the Institutional Review Board of the Japanese Red Cross Nagoya Daini Hospital, because no patient identifiers were used. All methods were carried out in accordance with relevant guidelines and regulations.

## Supplementary Information

Supplementary Table S1.

## Data Availability

All data generated or analyzed during this study are included in this article. Further enquiries can be directed to the corresponding author.
