# Supplementary material for: Admission temperature of very low birth weight infants and outcomes at three years old
Source: Sci Rep. 2022 Jul 13;12:11912. doi: 10.1038/s41598-022-15979-w (PMC9279326; doi:10.1038/s41598-022-15979-w)
Supplement: Supplementary file 1 — Supplementary Table S1. [file 41598_2022_15979_MOESM1_ESM.docx]

| **Supplemental Table S1. Multivariate model 2 explaining death or neurodevelopmental impairment at three years of age** | | | | | |
| --- | --- | --- | --- | --- | --- |
|  |  | Odds ratio | | |  |
|  |  | Mean | 95% CI | | p |
|  |  |  | Lower | Upper |  |
| Gestational age in weeks |  | 0.810 | 0.717 | 0.916 | <0.001 |
| Female sex |  | 0.486 | 0.242 | 0.975 | 0.042 |
| Antenatal corticosteroid |  | 0.734 | 0.359 | 1.500 | 0.397 |
| 5-min Apgar score <5 |  | 0.820 | 0.271 | 2.482 | 0.725 |
| Admission temperature (°C) |  | 0.441 | 0.261 | 0.746 | 0.002 |
| Intraventricular hemorrhage* |  | 11.052 | 2.636 | 46.323 | 0.001 |
| Culture pisitive sepsis |  | 1.724 | 0.583 | 5.102 | 0.325 |
| Neurodevelopmental impairment is defined as a performance developmental quotient of <70. | | | | | |
| *Grade III/VI by Papile’s definition. | | | | | |

An alternative model to explain the primary outcome with the adjustment for gestational age, sex, antenatal steroid use, Apgar score, severe intraventricular hemorrhage, and culture positive sepsis.
